# Response prediction in patients with gastric and esophagogastric adenocarcinoma under neoadjuvant chemotherapy using targeted gene expression analysis and next-generation sequencing in pre-therapeutic biopsies

**DOI:** 10.1007/s00432-022-03944-z

**Published:** 2022-03-05

**Authors:** Karsten Kleo, Vladimir M. Jovanovic, Alexander Arndold, Annika Lehmann, Hedwig Lammert, Erika Berg, Hannah Harloff, Christoph Treese, Michael Hummel, Severin Daum

**Affiliations:** 1 Institute of Pathology, Charité – Universitätsmedizin Berlin, corporate member of Freie Universität Berlin and Humboldt-Universität zu Berlin, Chariteplatz 1, 10117 Berlin, Germany; 2 Medical Department, Division of Gastroenterology, Infectious Diseases and Rheumatology, Charité Universitätsmedizin Berlin, Campus Benjamin Franklin, Hindenburgdamm 30, 12200 Berlin, Germany; 3 Experimental and Clinical Research Center, Charité University Medicine, Berlin and Max-Delbrück-Center for Molecular Medicine in the Helmholtz Association, Chariteplatz 1, 10117 Berlin, Germany; 4 Core Facility Genomics, Berlin Institute of Health at Charité – Universitätsmedizin Berlin, Charitéplatz 1, 10117 Berlin, Germany; 5 Institute of Informatics, Bioinformatics Solution Center, Freie Universität (FU), Takustr. 9, 14195 Berlin, Germany

**Keywords:** Gastric and esophagogastric adenocarcinoma, Perioperative chemotherapy, Histopathological response according to Becker score, Predictive biomarker in pre-therapeutic biopsies, Single nucleotide variants (SNV), copy number variations (CNV), gene expression

## Abstract

**Objectives:**

Perioperative chemo-(radio-) therapy is the accepted standard in European patients with locally advanced adenocarcinoma of the esophagogastric junction or stomach (AEG/AS). However, 30–85% of patients do not respond to this treatment. The aim of our study was the identification of predictive biomarkers in pre-therapeutic endoscopic tumor biopsies from patients with histopathologic response (Becker-1) versus non-response (Becker-2/3) to preoperative chemotherapy.

**Methods:**

Formalin-fixed paraffin-embedded biopsies from 36 Caucasian patients (Becker-1 *n* = 11, Becker-2 *n* = 7, Becker-3 *n* = 18) with AEG/AS, taken prior to neoadjuvant chemotherapy were selected. For RNA expression analysis, we employed the NanoString nCounter System. To identify genomic alterations like single nucleotide variants (SNV), copy number variation (CNV) and fusion events, we used Illumina TST170 gene panel. For HER2 and FGFR2 protein expression, immunostaining was performed. Furthermore, we analyzed the microsatellite instability (MSI) and Epstein–Barr virus (EBV) infection status by EBER in situ hybridization.

**Results:**

Heat map and principal component analyses showed no clustering by means of gene expression according to regression grade. Concerning two recently proposed predictive markers, our data showed equal distribution for MSI (Becker-1: 2; Becker-2: 1; Becker-3: 3; out of 29 tested) and EBV infection was rare (1/32). We could not reveal discriminating target genes concerning SNV, but found a higher mutational burden in non-responders versus responders and fusion (in 6/14) and CNV events (in 5/14) exclusively in Becker-3.

**Conclusions:**

Although we could not identify discriminating target genes, our data suggest that molecular alterations are in general more prevalent in patients with AEG/AS belonging to the non-responding Becker group 3.

**Supplementary Information:**

The online version contains supplementary material available at 10.1007/s00432-022-03944-z.

## Introduction

Adenocarcinoma of the esophagogastric junction or stomach (AEG/AS) is still one of the leading causes of cancer-related death worldwide (Ferlay et al. 2015; Kowollik 2020). Unfortunately, most AEG/AS are asymptomatic at the beginning of the disease and therefore often diagnosed at an advanced stage. The efficacy of perioperative chemotherapy has been proven for patients with locally advanced AEG/AS in several studies (Magic/ FFCD/ FLOT4) and also in one study for preoperative chemoradiotherapy with the use of the CROSS-protocol in AEG (Al-Batran et al. 2019; Al-Batran et al. 2017; Cunningham et al. 2006; Oppedijk et al. 2014; Shapiro et al. 2015; Hagen et al. 2012; Ychou et al. 2011). However, a group of preoperative chemo-(radio-) therapy treated AEG/AS patients show only minor histologic response to neoadjuvant chemotherapy and these suffer from a dismal prognosis (Becker et al. 2003). Histologic response toward preoperative chemotherapy has been classified according to Becker et al. (2003) into responders (Becker-1) and non-responders (Becker-2/3). The histologic pathological regression score according to Becker has found introduction into prospective studies as not being influenced by consecutive therapy lines or individual treatment factors (Al-Batran et al. 2019).

However, neither predictive biomarkers nor Pet–CT showed reliable robust results for prediction of preoperative chemoradiotherapy in AEG/AS patients (Tao et al. 2015). Only HER2 overexpression has been shown to be predictive with trastuzumab treatment in stage IV disease (Bang et al. 2010). In HER2-positive gastric cancer, trastuzumab in combination with chemotherapy leads to an increase in overall survival compared to chemotherapy alone (Gong et al. 2016; Ryu et al. 2015; Soularue et al. 2015; Thuss-Patience et al. 2017). Immune checkpoint inhibition in patients with expression of PD-L1 and PD1 correlates with a better outcome. This led to the approval of pembrolizumab and nivolumab in patients with advanced stage AEG/AS (Lu et al. 2019; Kato et al. 2020). Molecular classification of gastric and esophagogastric cancer into four subtypes, (EBV)-positive, microsatellite instability (MSI), genomically stable (GS) and chromosomal instability (CIN) type, was presented in 2014 (Bass et al. 2014). However, relevant clinical implications except the above-mentioned improved response according to PDL1 expression as well as MSI with use of immune checkpoint inhibition are missing.

Recently, the role of MSI had come into discussion, initially presented by a Japanese group already in 2009 and European data in 2017, which showed that patients with MSI tumors had a survival disadvantage under neoadjuvant chemotherapy in contrast to patients with microsatellite stable tumors (Smyth et al. 2017; Yashiro et al. 2009). As an underlying mechanism, a decreased chemosensitivity, possibly due to other mutations in genes that may otherwise sensitize tumor cells to platin compounds, was discussed (Oki et al. 2009; Velzen et al. 2020). However, consecutive presented data did not confirm the initial findings (Haag et al. 2019; Kohlruss et al. 2019).

**Fig. 1:**
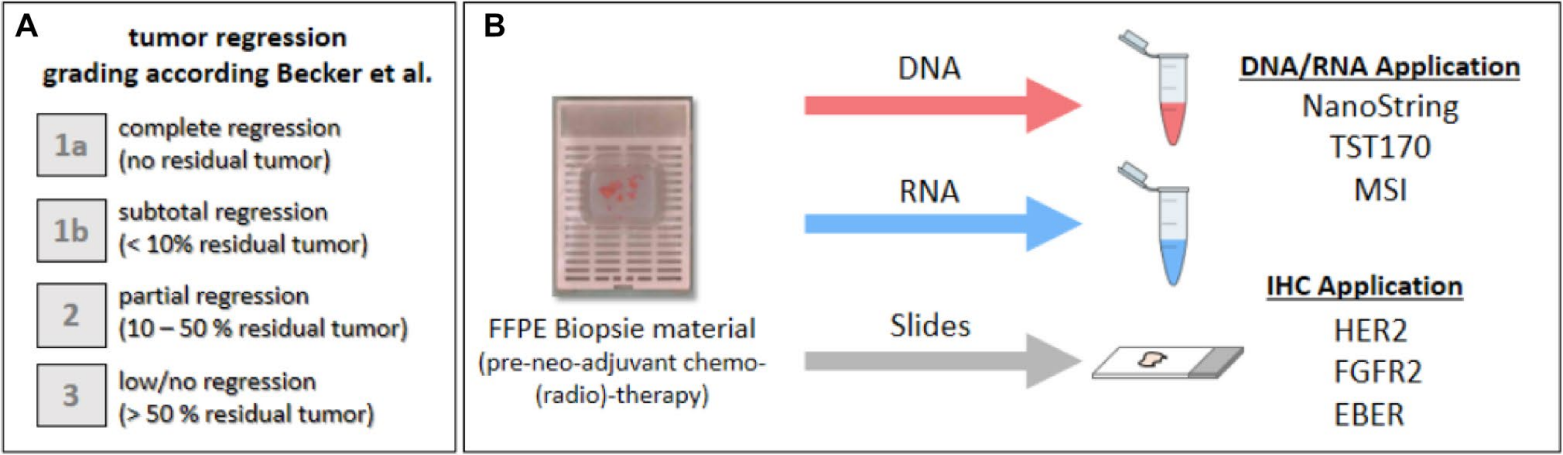
Analysis workflow. **A** Tumor regression classification system according to Becker et al. (2003). **B** Illustration of sample preparation and subsequent investigation from FFPE biopsies taken before therapy initiation

In this work we analyzed pre-therapeutic biopsy material from a collective (*n* = 36) of well-characterized patients with AEG/AS treated with preoperative chemotherapy and with different clinical outcomes to identify molecular markers for treatment prediction. (Fig. 1).

## Materials and methods

### Patient and sample collection

Formalin-fixed paraffin-embedded (FFPE), pre-therapeutic biopsies from 36 Caucasian patients diagnosed 2009–2016 for AEG/AS with different histopathological regression grades according to Becker et al. (2003) (Becker-1, *n* = 11; Becker-2, *n* = 7; Becker-3, *n* = 18) toward completed neoadjuvant FLOT or FLOT-like (oxaliplatin substituted by cisplatin; 5-FU substituted by capecitabine) chemotherapy were included in this study (Table 2). Patients initial clinical stage was ≥ cT2 or/ and cN + cM0. The tumor cell content of the pre-therapeutic biopsy samples was between 10 and 90% and no microdissection was performed. The study was approved by the Local Ethics Committee of the Charité—Universitätsmedizin Berlin (Approval No. EA4/075/15).

### Nucleic acid extraction

Genomic DNA and total RNA was extracted from FFPE biopsies employing the Maxwell 16 System DNA IQ Casework Pro Kit or, respectively, Maxwell 16 System RNA IQ Casework Pro Kit, each according to the manufacturer’s protocols (Promega Corporation, Madison, USA) as previously described (Bonnet et al. 2018; Vlahovic and Kubat 2012). Extracted DNA and RNA were quantified with the Qubit System using the Qubit DNA Assay or, respectively, Qubit RNA Assay (ThermoFisher Scientific).

### Epstein–Barr virus-encoded RNA in situ hybridization and immunohistochemistry

To detect Epstein–Barr virus (EBV)-encoded RNA (EBER), we performed an in situ hybridization (ISH) assay as already described (Anagnostopoulos et al. 1992; Fan and Gulley 2001). A ready-to-use EBER-ISH probe (BOND #PB5089) was used together with the Leica Bond-maX autostainer (Leica Biosystems, Illinois, USA) according to the standard BOND protocol. Immunohistochemical staining (IHC) was performed using sections derived from FFPE primary tissue samples with the help of the Leica Bond-maX autostainer (Leica Biosystems, Illinois, USA) according to the manufacturer’s instructions. After heat-induced epitope retrieval, the sections were incubated with anti-HER2/neu (4B5) (VENTANA—#790-4493) or anti-FGFR2 antibody (Sigma Aldrich—#HPA035305). Horseradish peroxidase-labeled anti-rabbit using the Bond Polymer Refine Detection Kit (Leica Biosystems, Illinois, United States) was employed to convert the chromogen substrate. Staining was performed with appropriate positive and negative controls. Evaluation of staining intensity was performed by a trained pathologist according to four level scoring (0—no staining, 1—light staining, 2—moderate staining, 3—strong staining) as previously described (Jia et al. 2016).

### Microsatellite instability (MSI) analysis

For the detection of microsatellite instability, a fluorescent PCR-based assay (MSI Analysis System Version 1.2, Promega) was used according to the manufacturer's instructions. The MSI Analysis System included fluorescently labeled primers for co-amplification of seven markers including five mononucleotide repeat markers (BAT-25, BAT-26, NR-21, NR-24 and MONO-27) and two pentanucleotide repeat markers (Penta C and Penta D). Additionally, an Internal Lane Standard 600 (ILS 600, Promega) was used. In brief, 20 ng of genomic DNA was used for the PCR under following conditions: initial denaturation for 11 min at 95 °C and 1 min at 96 °C, ramp 100% to 94 °C for 30 s, ramp 29% to 58 °C for 30 s, ramp 23% to 70 °C for 1 min, for 10 cycles, followed by ramp 100% to 90 °C for 30 s, ramp 29% to 58 °C for 30 s, ramp 23% to 70 °C for 1 min, for 20 cycles, and final elongation at 60 °C for 30 min. The PCR products were separated by capillary electrophoresis using a 3500 Genetic Analyzer. Data were analyzed with the GeneMapperR software (Applied Biosystems) by comparing sample marker patterns with the pattern of a positive amplification control (K562 High Molecular Weight DNA). A shift of two or more mononucleotide markers was considered as MSI-high, a shift of one mononucleotide marker was considered as MSI-low.

### NanoString

RNA samples (*n* = 36) underwent target-specific expression analysis using the NanoString nCounter gene expression system (NanoString Technologies, Seattle, WA, USA) as has been described previously (Geiss et al. 2008; Perez et al. 2019; Daum et al. 2020; Kulkarni 2011). nCounter PanCancer Immuno Profiling Panel (Cesano 2015) and the PanCancer Pathway Panel (Omarini et al. 2018) were employed to assess the expression level of a total of 1390 genes, including endogenous controls. The nCounter assay analysis was performed as previously described using 300 ng of total RNA, based on the manufacturer’s protocol (Kulkarni 2011).

### TruSight tumor 170 next-generation sequencing

Next-generation sequencing (NGS) was performed to identify genomic aberrations within genes of TruSight Tumor 170 (TST170) DNA and RNA assay (Illumina, Catalog no. 20028821). The enrichment-based targeted TST170 panel covers the coding regions of 170 tumor hot spot genes and allows the identification of SNPs, CNVs, and fusion genes. For TST170 analysis, DNA and RNA samples from the same tissue samples were used resulting in a total 24 biopsy samples (Becker-3, *n* = 14; Becker-2, *n* = 4; Becker-1, *n* = 6).

For this purpose, DNA and RNA concentrations were measured by fluorometric quantification on Qubit 3.0 (Thermo Fisher Scientific, Inc.) utilizing the Qubit Broad Range DNA assay and the Qubit Broad Range RNA assay (Thermo Fisher Scientific, Inc.), respectively. The quality of the total RNA was assessed by analyzing all samples on a TapeStation 4200 system with the RNA ScreenTape (Agilent Technologies) and the DV200 value was calculated for all samples. Only samples resulting in DV200 values of ≥ 20% were included in the library preparation process. RNA was diluted to 7 ng/µl in nuclease-free water and 8.5 µl of diluted RNA was processed according to the manufacturer’s recommendations. Genomic DNA was diluted to 7 ng/µl in TEB (Illumina, Inc.) and 12 µl of diluted samples were further diluted with 40 µl TEB for fragmentation. All DNA samples were fragmented to 90–250 bp (with a peak at ~ 125 bp) in microTUBE-50 AFA Fiber Screw-Cap tubes (PN: 520166; Covaris, Ltd.) using the Covaris ME220 system (Covaris, Ltd.) with the following settings: 75 W peak incident power, 25% duty factor, 1000 cycles/burst, 225 s. Further sample processing was performed for fragmented genomic DNA and purified cDNA simultaneously according to the instructions of the manufacturer. Prior to normalization, libraries were quantified by Qubit 3.0 and the Qubit High Sensitivity DNA assay (Thermo Fisher Scientific, Inc.). Libraries with concentrations ≥ 3 ng/μl qualified for bead-based normalization and pooling as well as sequencing. A total of eight samples, eight RNA libraries and eight DNA libraries originating from the same FFPE tissue were sequenced on one high-output NextSeq 500 (Illumina, Inc.) paired-end run with 101 cycles per read (2 × 101) and 8 cycles per index read.

### Next-generation sequencing data analysis

TST170 Illumina data analysis was performed by using S*OPHiA GENETICS DDM* Software (version 5.7.8.-b214-76be23a/March 2020), Saint Sulpice, Switzerland. The bioinformatics pipeline is able to call variants (SNVs/INDELs) with frequencies above the cutoff settings (4% variant frequency). Furthermore the pipeline algorithm is able to estimate fusion genes and CNV events (only CNV above five were designated), which is based on the coverage levels across samples within the same batch. The pipeline retained filter settings was used and a read depth under 1500 reads was designated as a low covered hit. The software used the following database versions at the time of analysis: ClinVar v20200312; COSMIC v87; dbNSFP v2.9; dbSNP v151; ESP 5400; ExAC r0.3.1; G1000 v5.20130502; GISAID EpiCoV v2020512, GenomAD r2.1; JAX-CKB v20200821.

For a general overview of the mutation status and to identify potential classifiers between different responder and non-responder groups, a bioinformatic pipeline powered by Bioinformatics Solution Center (BSC) was used. Briefly, the in house pipeline starts with raw reads data (FASTQ files), followed by quality control (QC) and trimming step, hg19/GRCh37 mapping using hisat2 2.1.0 program and finishing with variant calling by freebayes 1.3.1 program (Garrison and Marth 2012; Kim et al. 2019a).

### Statistic

Although to the small number of cases in our collective, we performed a statistical analysis for different data sets. To evaluate the statistical significance of immunohistochemical staining intensity for FGFR2 and HER2 expression between Becker groups we using the *t* test with Welch's correction (two-tailed) for unpaired comparisons. A *p* value less than 0.05 were considered as significant.

The Nanostring RNA expression data set was used with nSolver Software 4.0 using the Advanced Analysis Version 2.0.115 provided by NanoString. To calculate differential expression patterns, the samples were grouped according to their Becker scores (fringe groups: Becker-1 vs. Becker-3). The *p* value calculation was performed without adjustment and statistical significance was accepted at *p* < 0.01.

To determine differences within the total number of detected SNV (TST170 Data set), we performed in addition a Kruskal–Wallis test (Kruskal and Wallis 1952). It has to be noted that *p* value adjustment by false discovery rate procedure of Benjamini and Yekutieli was renounced, because of the small number of samples within this collective.

## Findings

**Fig. 2:**
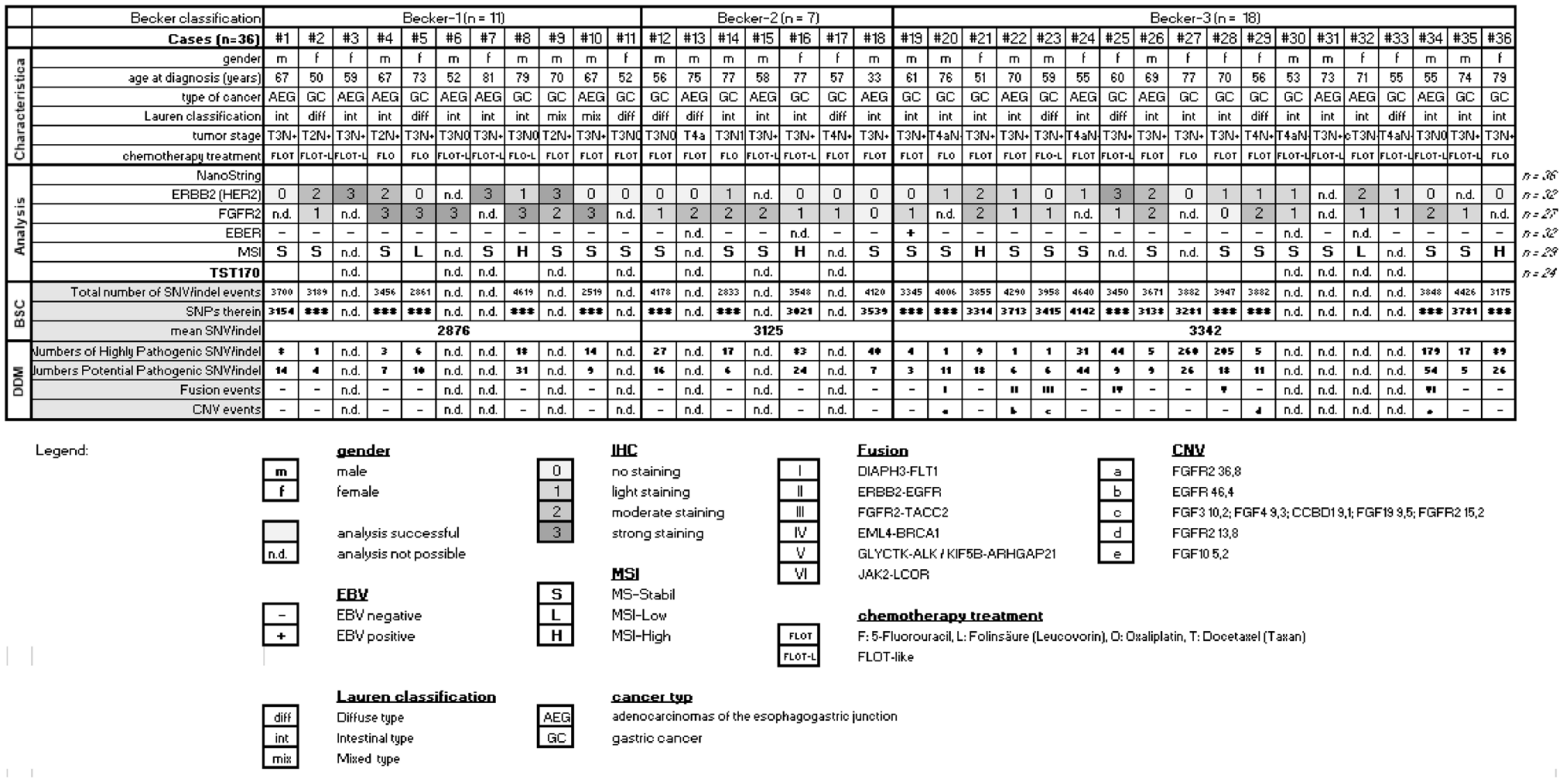
Analysis summary. Overview of the available samples employed for various methods. The gender (m/f) as well the histopathological tumor regression (Becker-1, -2 and -3) after neoadjuvant chemotherapy (FLO/T) in gastric adenocarcinomas according to Becker are illustrated. Immunohistochemical (IHC) staining results for HER2, FGFR2, status of microsatellite instability (MSI) as well as in situ hybridization for Epstein–Barr virus (EBV)-encoded RNA (EBER) are illustrated. Total numbers of single nucleotide variant (SNV) such as insertion and deletion (indel) events are carried out by the help of Bioinformatics Solution Center (BSC) using the TST170 data set. The variant classification according to pathogenicity was performed with help of SophiaGenticsDDM Software Version 5.7.8.-b214-76be23a (abbreviated in the figure is DDM). Total number pathogenic SNV/indel events and copy number variation (CNV), passing the filter setting SNV allele frequency > 4%, CNV > 5 and total read depth > 1500 reads, are illustrated. n.d. indicate that analysis was not possible from this case, because of insufficient material

We have carried out different types of analyses employing 36 pre-therapeutic biopsies of AEG/AS patients. After neoadjuvant chemotherapy, they were classified into Becker groups according to their treatment response as previously described (Becker et al. 2003). An overview of some clinical characteristics as well as an analysis summary are presented in Fig. 2. All analyses (IHC and NGS) were performed using the pre-therapeutic biopsies.

### Protein expression

Different immunohistochemical (IHC) staining patterns were detected between the investigated Becker groups. FGFR2 expression appears stronger in Becker-1 patients (responder) (5/7 cases stained score 3 and 1/7 cases stained score 2 and 1/7 cases stained score 1) compared to Becker-2 (3/6 cases stained score 2 and 3/6 cases stained score 1) and Becker-3 (4/14 cases stained score 2 and 8/14 cases stained score 1 and 4/14 cases stained score 0) patients (non-responder) (*p* < 0.0039).

HER2 expression did not show a clear delineation between the Becker groups or responder vs. non-responder group. A broad range of strong (score 3 and 2) and low (score 1 and 0) HER2 expressions are present in the entire collective (*p* < 0.2026).

We also determined the MSI and EBV status among the Becker groups and observed a random distribution among the patients (Fig. 2). However, both are rare events, only 1 of 32 investigated pre-therapeutic biopsies were EBV positive and only 4 of 29 were MSI-high.

### DNA alteration

As expected, analysis of the TST170 gene panel data revealed that individual molecular events are unable to predict the treatment response in pre-therapeutic biopsies. However, the total number of detectable SNV within the Becker-3 group is significantly higher compared to Becker-1 (*p* < 0.1818) (Fig. 2). Interestingly, we identified 7 different CNV events affecting the genes for FGFR2, EGFR, FGF3, FGF4, CCBD1, FGF19 and FGF10, which are also exclusively present in Becker-3 patients (f5 out of 14). This tendency of accumulation of genomic events in patients of the Becker-3 group (seven fusion events in six patients: DIAPH3-FLT1, ERBB2-EGFR, FGFR2-TACC2, EML4-BRCA1, GLYCTK-ALK, KIF5B-ARHGAP21 and JAK2-LCOR) was furthermore demonstrated for chromosomal translocations as depicted by RNA-NGS for the detection of the corresponding fusion transcripts (Fig. 2).

**Fig. 3:**
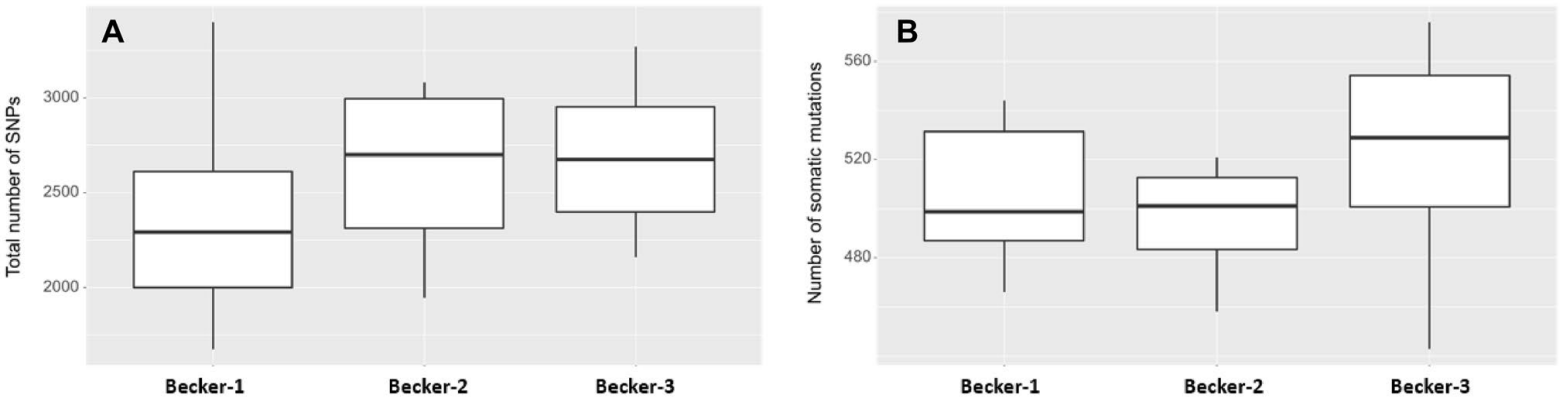
Total numbers of (**A**) single nucleotide polymorphism (SNP) and (**B**) somatic mutations. Due to the small number of cases of Becker-1 (*n* = 6), Becker-2 (*n* = 4) and Becker-3 (*n* = 14) no statistically significant (*p* < 0.05) difference between the mean values of Becker groups was found by pairwise Wilcoxon rank-sum tests (**A** Becker-1 vs. Becker-2: *p* value = 0,47; Becker-1 vs. Becker-3: *p* value = 0,23; Becker-2 vs. Becker-3: *p* value = 0,79 and **B** Becker-1 vs. Becker-2: *p* value = 0,76; Becker-1 vs. Becker-3: *p* value = 0,23; Becker-2 vs. Becker-3: *p* value = 0,12)

**Fig. 4:**
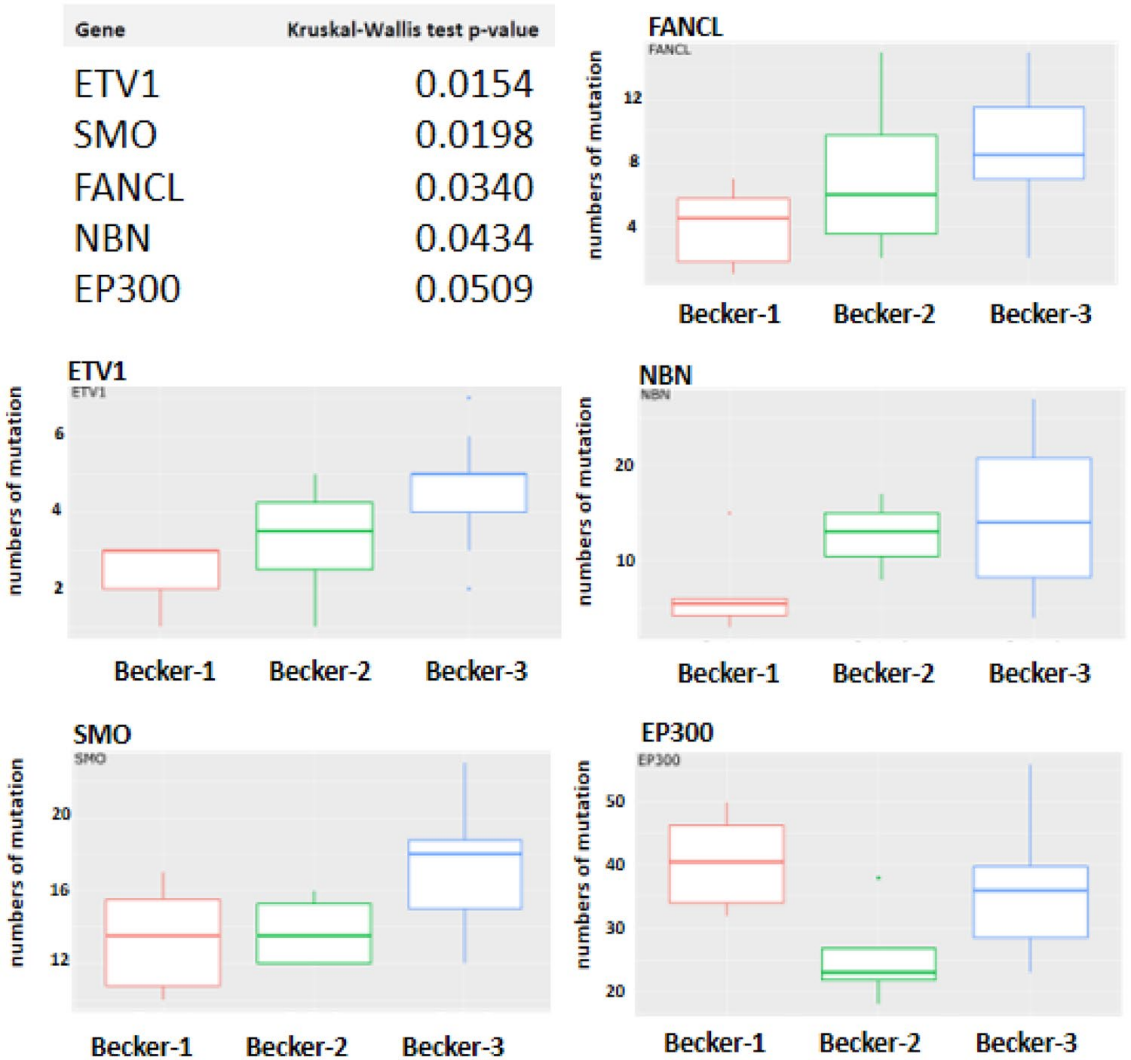
Numbers of mutation in hotspot genes (ETV1; SMO; FANCL; NBN and EP300) between Becker groups (Becker-1, Becker-2 and Becker-3) are presented together with unadjusted *p* values of Kruskal–Wallis test

To see if any gene is more frequently mutated in one of the Becker groups, the total number of SNV per gene was calculated within each sample and within the groups (Fig. 3). Top mutated genes were ETV1, SMO, FANCL, NBN and EP300 the least represented genes were MYD88, MCL1 and CEBPA (data not shown). In Fig. 4 the top five genes are given together with unadjusted *p* values and the total counts of SNPs in these genes were shown.

To have a general overview of similarity or clustering/stratification among individual genotypes, the principal component analysis (PCA) was undertaken in PLINK 1.9 software. Top principal components were extracted from the variance-standardized relationship matrix of genotypes. Genotype was called for all SNV loci, imputed as homozygous reference allele when alternative allele was not called in a sample. However, no clustering of samples according to Becker grouping was identified, but only (small) deviations of several samples from the central cluster cloud could be detected (Appendix Fig. 8).

**Fig. 5:**
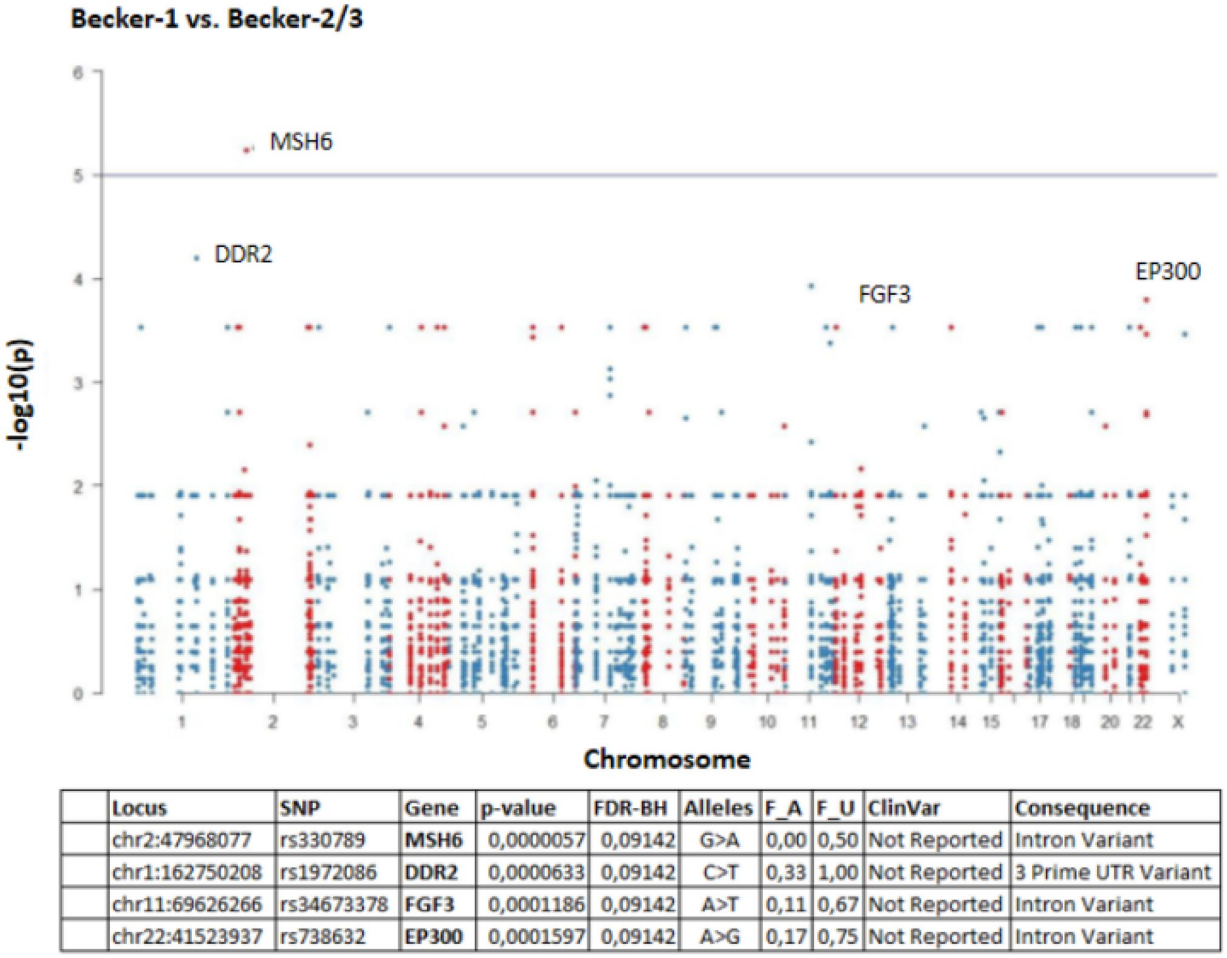
Manhattan plots of association analysis of SNPs between Becker-1 and Becker-2/3 groups. Table below shows identified different intronic SNPs. SNP—published variant identifier; gene—genomic content; alleles—major > minor alleles; F_A—minor allele frequency among Becker-2/3 samples; F_U—minor allele frequency among Becker-1 samples; ClinVar—interpretation of effect in NCBI ClinVar database

For comparison between Becker groups, the variant call files (VCF) were merged, and only biallelic SNV loci were kept. Total numbers of loci that were used and association between SNPs and Becker groups was calculated in PLINK by 1df Chi-square allelic test (standard case/control association analysis). The results of association of alleles with specific Becker groups are shown in Fig. 5. The Manhattan plots yield four significant SNP locus (MSH6, DDR2, EP300 and FGF3 gene) that differ between responder (Becker-1) and non-responder (Becker-3) group (Fig. 5).

### RNA expression

**Fig. 6:**
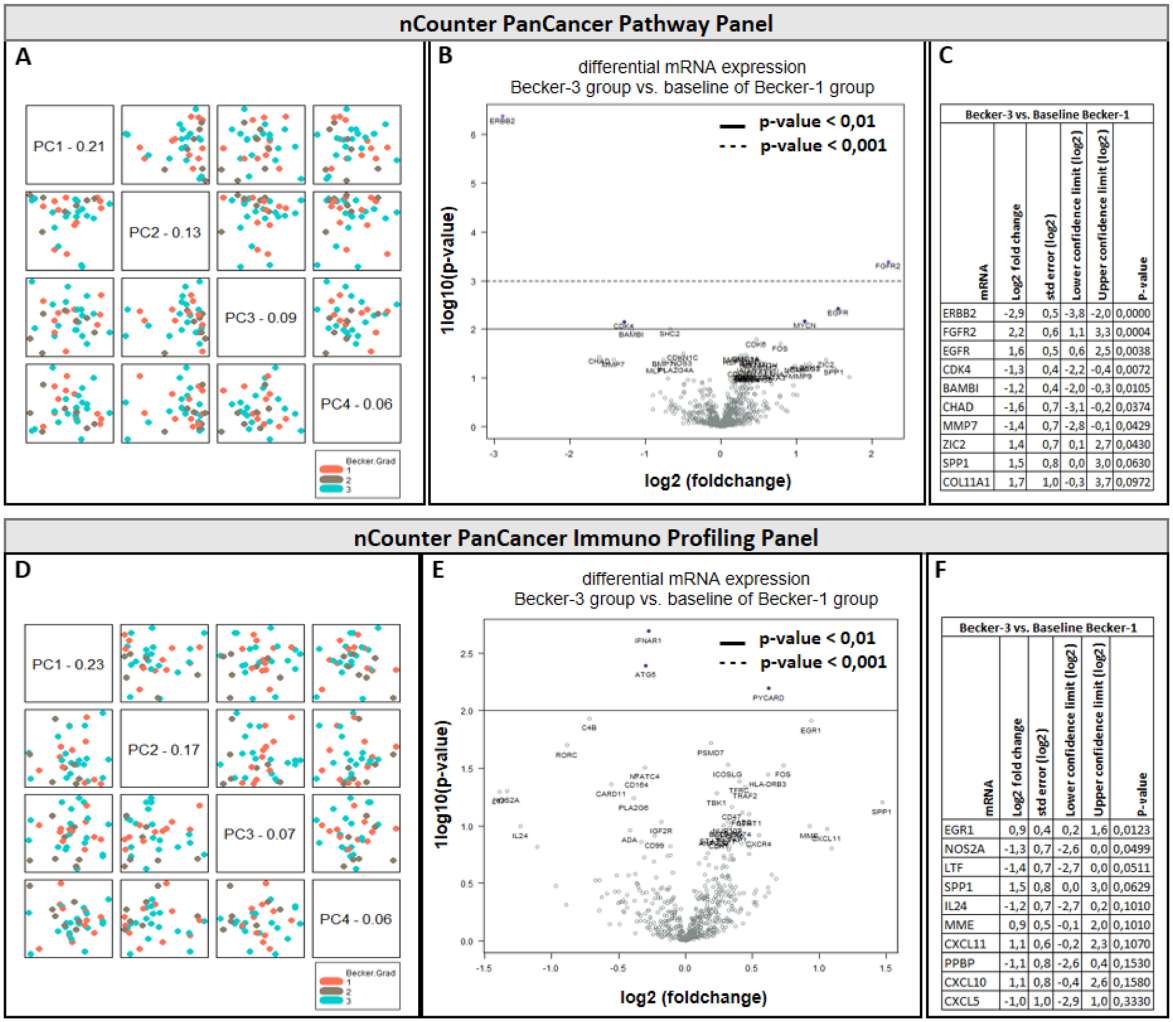
NanoString results using nCounter PanCancer Pathway Panel (**A**–**C**) and nCounter PanCancer Immuno Profiling Panel (**D**–**F**). (A/D) Principal component analysis (PCA) maps of all data. (B/E) Volcano plot displaying differential expressed genes -log10 (*p* value) and log2 fold change between responder (Becker-1; *n* = 11) and non-responder (Becker-3; *n* = 18) group. (C/F) Tabular extract of the highest log2 fold changes within the set of investigated target genes of nCounter corresponding panel

For RNA expression, we performed a target-specific analysis using the NanoString nCounter platform. Two panels (PanCancer Pathway Panel and PanCancer Immuno Profiling Panel) consisting of a total of 1390 target genes were used to identify differentially expressed genes (Fig. 6). Principal component analysis (PCA) demonstrates no clustering (Fig. 6A, D). Among the investigated genes no significant differential expression was observed between responder (Becker-1) and non-responder (Becker-3) groups (Fig. 6B, E). Top differentially expressed genes are summarized in Fig. 6C, F. However no significant differences in RNA expression were observed, rather the higher ERBB2 expression (Log2 fold change 2,9) in Becker-1 group was affected by high ERBB2 expression in two individual Becker-1 samples.

To get a general impression of RNA expression within the whole collective, we downsampled the NanoString data to 24 samples, and expression analysis in the DESeq2 package (in R 4.0.2) was done for NanoString (770 genes) data and TST170 (55 genes) data. The matrix of NanoString and TST170 experiment raw expression data were imported to R for DESeq2 analysis. DESeq2 statistical model internally corrects/normalizes the data (Love et al. 2014).

**Fig. 7:**
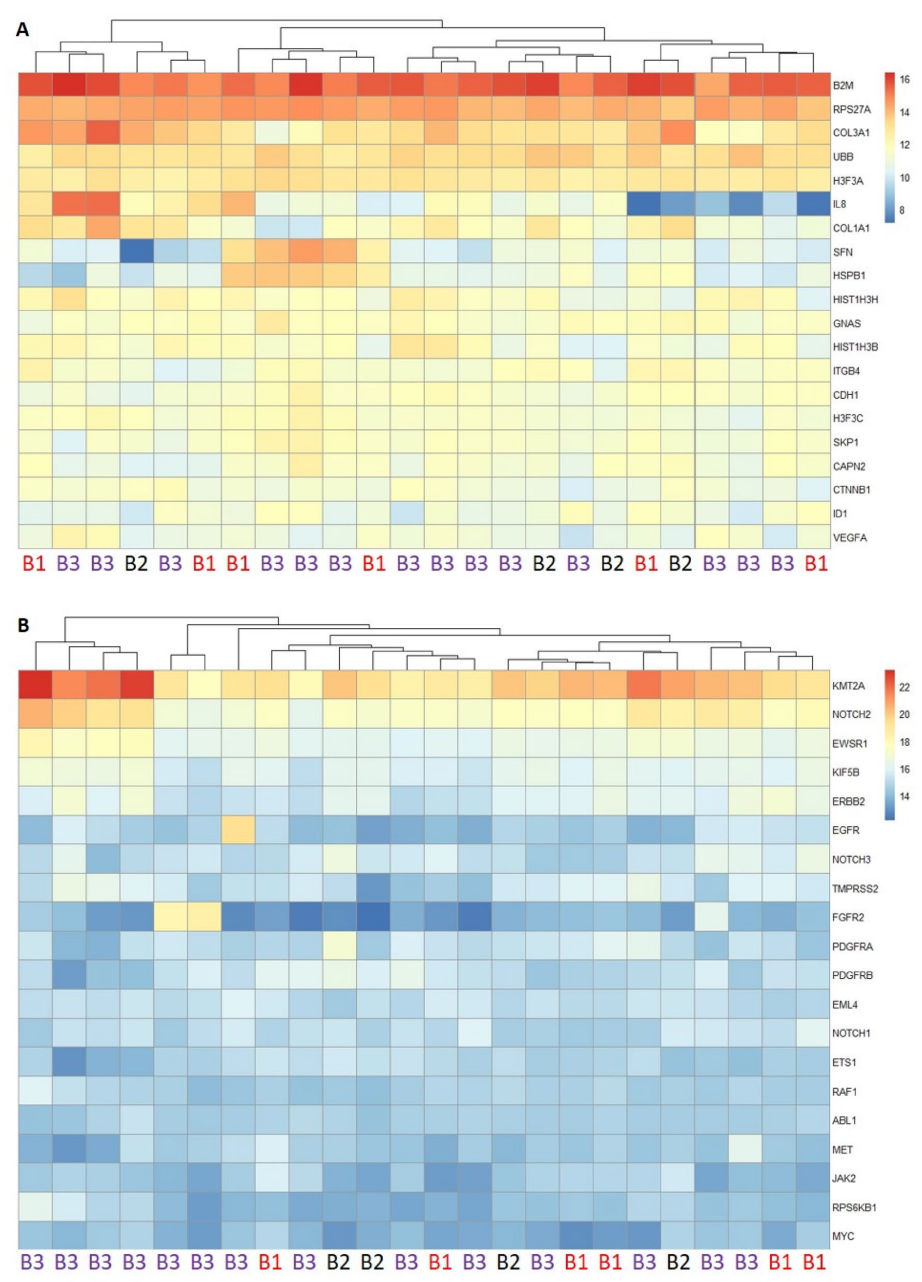
Heatmap of 20 highest expressed genes in NanoString (**A**) and TST170 (**B**) experiment among 24 samples representing Becker groups (B1–B3)

To explore the count matrix, the top 20 expressed genes heat map was produced with the clustering of samples for NanoString Data Set (Fig. 7A) and TST170 Data Set (Fig. 7B). Similar to the previous analysis in nSolver software by NanoString, clusters did not represent Becker groups. However, it can be seen that in B2M-, RPS27A-, KMT2A and NOTCH2-gene represent the highest expressed genes within the whole collective.

## Discussion

In this study based on a thorough analysis of molecular alterations in opposed responding groups of adenocarcinoma of the esophagogastric junction or stomach (AEG/AS) to neoadjuvant chemotherapy, we could show that SNV, CNV und fusion events were accumulated in the worse responder group Becker-3. The most accepted classification system of these kinds of carcinomas is the AEG classification system according to Siewert based on the tumor location and was supplemented via the Cancer Genome Atlas (TCGA) by non-clinical features such as Epstein–Barr virus infections, microsatellite instability status or PD-L1 and PD-L2 amplification (Cancer Genome Atlas Research et al. 2014; Siewert and Allgöwer 2001; Cancer Genome Atlas Research et al. 2017). TCGA classification includes four groups: EBV-associated tumors (2–5%), tumors with MSI (about 10%), chromosomal instability (CIN) (about 40%) of patients and comprise the histological intestinal group, and the genomic stable (GS) group of tumors which harbors the diffuse histological type and is associated with CDH1 mutations (about 40%). Clinical data, acquired in the French Prodige Group and the German AIO group, were negative for response discrimination concerning patients with diffuse (GS) versus intestinal type (CIN) (Al-Batran et al. 2019; Al-Batran et al. 2017; Cancer Genome Atlas Research et al. 2014, 2017; Eveno et al. 2019). Other robust predictive markers have not been found concerning the GS and CIN group.

In Caucasian patients data on pharmacogenetics (metabolism of 5-FU/oxaliplatin) (Smyth et al. 2017; Ott et al. 2006), tumor suppressor gene p53 and the transcription factor NF-kB, the cell cycle regulating protein p21, expression of tyrosine kinases (EGFR, Her2, c-Met) was associated with prognostic and/or predictive impact (Abdel-Latif et al. 2004; Nakamura et al. 2004; Ruhstaller et al. 2018; Yamamoto et al. 2017) However, these were often not done on pre-therapeutic biopsies or further confirming data were missing (Smyth et al. 2017; Stahl et al. 2018). Therefore, the identification of predictive markers in pre-therapeutic endoscopic tumor biopsies still is one of the urgent questions in upper gastrointestinal oncology and has been summarized in an overview recently (Gervaso et al. 2021). Thus, we investigated the molecular background of 36 pre-therapeutic biopsies of AEG/AS patients, with opposite histopathologic response to standard neoadjuvant chemotherapy, for predictive molecular markers.

Initially, we performed immunohistochemical staining for Her2 and FGFR2 protein expression using neoadjuvant biopsies of AEG/AS patients. Pre-therapeutic endoscopic tumor biopsies from patients with histopathologic response (Becker-1) showed increased FGFR2 expression by immunohistochemistry, whereas no Becker-2/3 sample (*n* = 20 with stainable tissue) showed strong staining expression (score 3) of FGFR2. Mutational activation and consecutive overexpression of FGFR2 have been associated with an advanced stage at diagnosis and decreased overall survival in several studies with Asian focus (Hierro et al. 2017; Kim et al. 2019b; Klempner et al. 2019). Furthermore FGFR2 fusions/rearrangements events have demonstrated their clinical relevance in cholangiocarcinoma patients, which benefit from treatment with selective FGFR1-3 inhibitors. Mostly, FGFR-overexpression was associated with diffuse type of GC (genomically stable tumors according to TCGA), but the relevance of these data is unclear (Inokuchi et al. 2017).

While immunohistochemistry showed no different distribution between Becker-1 and Becker-2/3 samples, Her2 (ERBB2) RNA was overexpressed in Becker-1 samples. However, this overexpression could be relocated to two single samples with exceptionally increased expression. Our data go along with a meta-analysis on the prognostic value of Her2 in gastric cancer. The authors concluded on the basis of studies conducted worldwide that Her2 overexpression is of negative prognostic relevance and is associated with intestinal type, more advanced disease and male gender (Lei et al. 2017). However, in a small neoadjuvant study conducted in Italy with use of epirubicin, the Her2 overexpressing group showed a significant improved overall survival, which points to several unsolved questions in understanding Her2 mechanisms (Personeni et al. 2017).

Epstein–Barr virus (EBV) infection (EBER expression) is a very rare event in our cohort of patients, since only 1 patient out of our 32 tested was positive. Our finding is very much in line with published data where only 6 out of 143 patients with neoadjuvant chemotherapy and pre-therapeutic biopsies displayed EBV-positivity with no significant correlation with tumor regression (Cancer Genome Atlas Research et al. 2014, 2017).

In harmony with published data, only a small proportion (4/32) of our AEG/AS patients were associated with MSI, however, with no preference to one of the Becker groups. To this end, we cannot confirm MSI as a negative predictive factor for the response to preoperative chemotherapy and preexisting data are inconsistent. In general, patients with MSI show an improved survival over all stages (Polom et al. 2018); however, predictive data for chemotherapy response are missing or contradictory. In 2017, the Magic group published data demonstrating a negative effect of preoperative chemotherapy in MSI patients with limited patient numbers (Smyth et al. 2017). Although these data have been revised through other groups with higher patient numbers, these analyses were based on retrospective data (Kohlruss et al. 2019; Kohlruss et al. 2021). Female gender might be a positive predictive marker in combination with MSI in neoadjuvant chemotherapy setting (Kohlruss, et al. 2021). Altogether, published data and our own limited observations allow the conclusion that EBV and MSI status cannot be used as solid biomarkers for a therapy response prediction in AEG/AS patients.

Furthermore, we investigated DNA alterations and RNA fusion transcripts using the TST170 gene panel and its RNA fusion component for our cohort. The most commonly identified SNV was the FGFR4 polymorphism (Exon 9: c.1162G > A; p.(Gly388Arg), which was detected in 15 out of 24 samples. All detected SNVs are summarized in supplementary table 1. Since we could not identify any SNV event, which is exclusively usable to separate responder from non-responders, we broadened our view and looked at the general burden of mutation (BoM) as well as the genes that were most affected by a genetic aberration. Our data suggest that the non-responder fraction (Becker-3) is much more often affected by genomic aberration and shows a higher burden of mutation compared to the responder fraction (Becker-1). We identified five hot spot genes (ETV1; SMO; FANCL; NBN and EP300) which showed differential molecular burden between Becker groups (Becker-1, Becker-2 and Becker-3). Except for EP300, the mutational burden is always shifted to the Becker-3 group. Chromosomal instability (CIN) of gastric and esophagogastric adenocarcinoma is already known (Cancer Genome Atlas Research et al. 2014, 2017) and studies suggested that CIN is associated with a poor clinical outcome in solid tumors (Carter et al. 2006; Walther et al. 2008). It is tempting to speculate that the higher molecular burden of mutation in non-responders could also be associated with a higher probability to form tumor-associated neoantigens. These patients could possibly benefit from therapy with immune checkpoint inhibitors. In addition, it is proposed to use the observation of tumor molecular burden (TMB) to potentially aid in the diagnostic assessment of therapy prospects.

Taken together, in this deeply investigated group we found that the total number of aberrant SNV, CNV und fusions events is accumulated in the non-responder group (Becker-3). Our work demonstrates the principle of using pre-therapeutic biopsies itself to support response prediction. It is recommended to expand this work to a larger collective and involve further diagnostic tools such as the tumor molecular burden (TMB) or homologous recombination deficiency (HRD) estimation.

Our work demonstrates the principle potential of using pre-therapeutic biopsies in the search of molecular markers to support response prediction, although we did not find a clear biomarker in this specific setting. It is planned to expand this approach, especially FGFR2 staining to a larger patient collective and involve other diagnostic tools such as the tumor molecular burden (TMB) or homologous recombination deficiency (HRD) estimation.

### Supplementary Information

Below is the link to the electronic supplementary material.

Supplementary file1 (XLSX 3049 KB)

## Figures and Tables

**Fig. 8:**
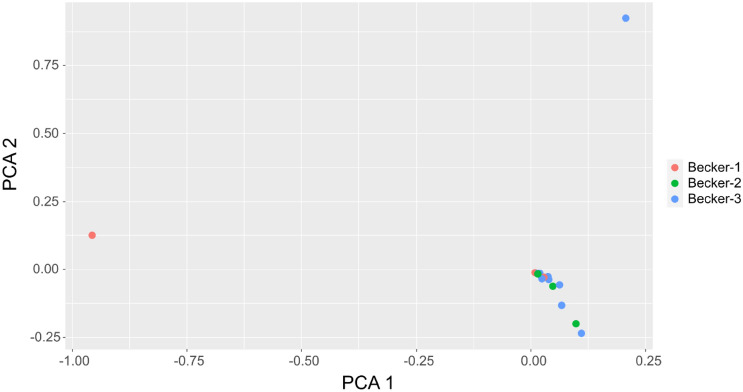
PCA plots of genotypes

## Appendix

See Fig. 8.
